# Commercial and public payer opioid analgesic prescribing policies: a case study

**DOI:** 10.1186/s13011-020-00340-z

**Published:** 2021-01-06

**Authors:** Cynthia L. Arfken, Victoria Tutag Lehr

**Affiliations:** 1 Department of Psychiatry and Behavioral Neurosciences, Wayne State University School of Medicine, 3901 Chrysler Drive, Suite 1B, Detroit, MI 48201 USA; 2 Department of Pharmacy Practice, Eugene Applebaum College of Pharmacy and Health Sciences, Wayne State University, 259 Mack Avenue, Room 4144, Detroit, MI 48201 USA

**Keywords:** Opioid prescribing, Commercial insurance, Public insurance, Policies, Regulations, Temporal trends

## Abstract

**Background:**

One strategy to address the high number of U.S. opioid-related deaths is to restrict high-risk or inappropriate opioid analgesic prescribing and dispensing. Federal and state laws and regulations have implemented restrictions but less is known about commercial and public payers’ policies aside from clinician anecdotal reports that these policies are increasing. To assess the number and types of policies with temporal trends, we examined commercial and public (Medicaid) payer policies in one state, Michigan, that has high opioid-related deaths and implemented opioid analgesic prescribing laws.

**Methods:**

Policies for seven large commercial payers and the public payer for 2012–2018 were reviewed and categorized by actions. Joinpoint regression was used to summarize temporal trends on number of policies for all payers and subgroups.

**Results:**

Across the 7 years, there were 529 action policies (75.57 (95% confidence intervals (CI) 35.93, 115.22) actions per year) with a range of 36 to 103 actions by payer. Limitations on number of days for initial prescriptions and prior authorizations were the most frequently implemented policy. The temporal trend showed a decline in new policies from 2012 to 2013 but a steady increase from 2014 to 2018 (average annual percent change or AAPC=29.6% (95% confidence intervals 13.2, 48.5%)). The public payer (*n*=47 policies) showed no increase in number of policies over time (AAPC=2.9% (95% CI -41.6, 61.6%).

**Conclusions:**

The eight commercial and public payers implemented many new policies to restrict opioid analgesic prescribing with a steady increase in the number of such policies implemented from 2014 to 2018. This case study documented that at least in one state with high opioid-related deaths and multiple commercial payers, new and different policies were increasingly implemented creating barriers to patient care. The impact of these policies is understudied, complicating recommendation of best practices.

## Introduction

Opioid-related deaths in the United States from prescription opioid analgesics is a public health problem with increases from 3442 deaths in 1999 to 17,029 in 2017 or a nearly 5-fold increase [1]. In 2018 the number of deaths declined to 14,975 [2], the first documented decline since the current mortality coding system was implemented in 1999. Although the number of deaths declined, too many people continue to die from accidental or intentional prescription opioid analgesic ingestion.

To address the high number of prescription opioid analgesic-related deaths, multiple strategies have been implemented to reduce unsafe prescribing and dispensing of opioids. At the federal level, actions include changing hydrocodone from Schedule III to the more restrictive Schedule II controlled substance classification [3]. At the state level, actions include implementing Prescription Drug Monitoring Programs (PDMP), an electronic database of prescription opioid analgesics and other controlled substance medications dispensed with related prescription-specific information and limited information on patient, prescriber and dispensing pharmacy [4, 5]. States varied in when they established a PDMP, type of information collected, and the requirements for use [6]. Several states including Michigan have integrated their PDMP systems into electronic health records and pharmacy dispensing systems [7]. States also vary in other initiatives such mandatory review by prescribers of patients receiving long-term opioid analgesic therapy [8] and type of user allowed to request and review prescription history reports under defined situations [9]. In 2012, access to the PDMP was extended to pharmacy benefit managers in several states including Michigan to allow opioid prescription utilization oversight for purposes of identifying fraud or misuse [10]. At the federal level, Medicare (the payer for adults ages 65 years and older and those with disabilities) has published new regulations for prescribing opioid analgesics annually since 2015; the regulations apply to all covered lives regardless of state of residence. If the state regulations or laws are more restrictive than those of Medicare, the prescriber must comply with those of the state. Commercial and state public (i.e., Medicaid) payers have also implemented opioid prescribing policies (note: we will use the term “policy” to refer to commercial payer policies and public payer regulations). Overall, with the exception of PDMP evaluations, reviews have highlighted the small number and low quality of published evaluations of state laws and regulations, and payer policies on opioid prescribing [11–13].

For the few payer policies evaluated, there have been comparisons between states (and over time) for prior authorization by Medicaid [14]. Policies of commercial payers have been mostly limited to pre and post implementation of providers’ behavior [15] or number of prescriptions [16]. In Massachusetts, Blue Cross Blue Shield implemented a comprehensive policy for opioid prescribing with seven different actions or components in 2012 [16]. The investigators showed a 14.7% decline in average monthly prescribing rate for all opioids during the 3 years post-implementation, but it is not clear that the decline was specific to their members or independent of other interventions such as education, media attention or policies of competing commercial payers. For example, two studies showed that policies can result in members dropping coverage [17] or paying cash for prescriptions [18]. Other complications include patients with more than one health plan coverage and prescribers paneled by more than one commercial payer and therefore exposed to multiple opioid prescribing policies. As stated by Comerci and colleagues [19]:

*Increasingly, prescription-drug plans are instituting complicated and confusing opioid-prescribing rules. Often, limits are placed on dosage forms, quantities, or both without any evidence that such restrictions will ameliorate opioid overuse and misuse. Navigating these rules is time consuming for both clinicians and pharmacists…*

Before the impact of payer policies can be evaluated, the magnitude of the “complicated and confusing” policies have to be articulated. The aim of this study is to quantify the number and type of opioid analgesic prescribing policies implemented by commercial payers and Medicaid in one state, Michigan. This approach allows us to explore the policies while holding constant state laws and regulations.

### Michigan as a case study

Michigan makes a reasonable case study as it was affected by the opioid epidemic with drug overdose deaths increasing from age-adjusted rate of 6.1 per 100,000 in 1999 to 21.7 in 2017 [20]. The age-adjusted rate then declined by 4.1% to 20.7 per 100,000 in 2018 with 78% involving at least one opioid [20]. In 2018 Michigan providers wrote 62.7 opioid prescriptions per 100 residents compared to U.S. rate of 51.4 prescriptions [20].

In Michigan, the number of opioid prescriptions paid by commercial insurance accounted for 62.1% of such prescriptions in 2015 with the total number of opioid prescriptions declining in subsequent years [21]. From 2015 to 2018, the number of prescriptions declined 30.5% for commercial payers, 11.4% for Medicaid, 15.2% for Medicare and 25.0% for cash [21]. The number of opioid prescribers also declined during this time period from 55,180 to 53,850 similar to national analysis [22] even though opioid prescriptions and prescribers were added with the phased inclusion of Veteran’s Administration prescription data into the PDMP [23]. The Veteran Administration prescribers were using Michigan’s PDMP by 2018 when prescribers and pharmacists were required to register [24].

Other changes include Michigan requiring Physician Assistants and Advanced Practice Nurses in 2017 to obtain their own Drug Enforcement Administration registration number instead of prescribing under a delegating physicians’ number [25]. The PDMP originally adopted in 2008 was replaced in 2017 with an updated version having electronic medical record interface and improved drug prescription history reporting capabilities. A proprietary patients’ overdose risk score was also added. In 2017, Michigan passed a law protecting pharmacists from civil liability if they refuse to dispense controlled substance prescriptions when they have a reasonable and good-faith belief that the prescription was not written by a prescriber in good faith or the prescription did not have a medical purpose [26].

In 2017 Michigan mandated prescribers and pharmacist to register and check the PDMP prior to prescribing or dispensing opioids and other controlled substances [25]. Additional laws applicable to all payers implemented during 2018 and 2019 were informed consent for opioid treatment for minors and providing patient information on opioid risks [27]; requiring a *bona-fide* provider-patient relationship for prescribing controlled substances [28] (implemented March 31, 2018 but later extended to March 31, 2019); and limit of 7 days prescribing of opioids for acute pain [29], (implemented July 1, 2018).

### Purpose

Quantifying of number and type of payer policy is a necessary first step in describing the opioid analgesic prescribing restrictions for clinicians and pharmacists prior to evaluating their policy impact. As such, our expectations were that all payers examined would have opioid analgesic prescribing policies, and consistent with the observation by Comerci and colleagues [19], more policies would be implemented over time.

## Methods

To quantify the number and types of polices, we reviewed and categorized policies implemented by both public (i.e., Medicaid) and major commercial insurers in Michigan on opioid prescriptions from January 2012 through December 2018. This time frame included the estimated 2012 peak Michigan opioid prescribing rate and subsequent monotonic decline [30]. We documented policy implementation by year to highlight trends and variability in policy activity by individual payers. Policies related to treatment of substance use disorder or naloxone access were excluded as the study focus was on opioid analgesic prescribing.

### Commercial payers

Commercial payers included in the study were Blue Cross Blue Shield of Michigan, Blue Care Network, Priority Health, Health Alliance Plan, Aetna, United Healthcare, and Cigna. They are for-profit except Health Alliance Plan (nonprofit), Blue Care Network and Blue Cross Blue Shield of Michigan (became a nonprofit mutual in 2014). During the 7-year period, Blue Cross Blue Shield of Michigan was the dominant commercial insurer in the state. In 2016 and 2017, Blue Cross Blue Shield of Michigan, Priority Health and Health Alliance Plan accounted for at least 80% of the large group commercial health insurer market in Michigan [31].

### Obtaining opioid prescribing policies

To obtain information on specific payer opioid policies, one investigator (VTL) searched the Michigan Department of Health and Human Services website for Preferred Drug List updates by year using the following search terms: “CNS medications-opioid analgesics”, “narcotic analgesics”, “opioid analgesics”, and “pain relievers-narcotics (or opioids)”. The Preferred Drug List is the formulary for Medicaid in Michigan. The Michigan Pharmacy and Therapeutics Committee meets to review and recommend changes to the Preferred Drug List at least quarterly. The individual commercial plan websites were also reviewed for formulary updates by year using the same search terms. Commercial payers’ formularies are reviewed at least annually by their pharmacy and therapeutic committees.

Once updates for Medicaid and commercial payers’ formularies were identified, the search turned to the policies underlying them. For Medicaid, the Pharmacy and Therapeutic committee’s publicly available quarterly meeting minutes were reviewed for prescribing policies. The individual commercial payers’ websites were reviewed for press releases and policy updates to explain changes. For the few cases where there were formulary updates without policy explanation, responsible individuals at the payer plans were contacted for clarification. The Michigan Pharmacist Association website was also independently searched for communications regarding opioid prescribing policies to minimize the risk of inadvertently omitting a policy. From this examination, a chronological list of policies for each payer across the 7-year study period was compiled.

### Categorizing policies

The formulary updates could include addition or removal of individual medications, and actions across multiple medications by dosage form (e.g., long acting opioids) or route (e.g., transmucosal fentanyl products). Other common formulary actions include time limitations, quantity limits, and prior authorizations for select medications.

To categorize the formulary updates the investigators started with a list of common formulary management strategies used by payers to promote safe and appropriate opioid prescribing [32, 33]. The individual components, or actions, of the strategies were then developed through discussion with five experienced pharmacists practicing formulary management, community, hospital, long-term care and home infusion. Importantly, an individual policy can result in more than one action. An example would be “lock-in program” initiated by a payer to restrict members’ access to opioid analgesics [18]. Lock-in programs identify members with pre-defined criteria and restrict their access to one prescriber and pharmacy for opioid prescription claims reimbursement [18]. We coded lock-in programs as 1) creating a patient registry, 2) limitation on providers, and 3) limitations on pharmacies. This coding system allows flexibility for different policies with overlapping actions.

During the initial review of policies, it became apparent that another action, “pharmacy safety review” or “step-edits”, was required, resulting in 13 separate actions (Table 1). The action of safety reviews indicates that the pharmacist must review and document approval to dispense medication. Information required for the pharmacy review can include patient must have first tried and failed other (often first-line and less expensive) therapies, demonstrated an intolerance/allergy/adverse reaction to first-line therapies, or require prescribing by a specialist provider. It also specifies criteria and actions for pharmacists to ensure that a medication is appropriate for an individual patient with respect to dosage, concurrent medications or other factors.

**Table 1:** Policy actions by seven commercial insurers and Medicaid-for-service for prescribing opioid analgesics by year, Michigan

Specific Action	2012	2013	2014	2015	2016	2017	2018	Total	# payers implemented
LIMITATIONS on number days initial prescrpiton	8	4	4	7	11	22	27	**83**	**8**
PRIOR AUTHORIZATION on initial prescription	9	7	5	9	13	16	18	**77**	**8**
Formulary limitation	5	1	3	5	10	12	24	**60**	**8**
Registry of patients	11	4	6	6	6	7	18	**58**	**8**
Pharmacy safety review/step edit	4	6	6	9	6	14	12	**57**	**8**
PRIOR AUTHORIZATION for long-acting/extended release opioids	6	2	5	4	3	6	10	**36**	**8**
LIMITATIONS on providers	4	6	5	7	3	8	3	**36**	**8**
LIMITATIONS on number refills	6	2	3	2	0	10	13	**36**	**7**
LIMITATIONS on dosages within formulary	4	1	2	2	5	7	9	**30**	**7**
PRIOR AUTHORIZATIONS for refill prescription(s)	1	3	2	1	1	8	9	**25**	**7**
PRIOR AUTHORIZATIONS for higher potency opioids	2	1	2	1	1	5	9	**25**	**8**
Feedback to providers on opioid prescribing	2	2	0	3	0	1	1	**9**	**3**
Incentives to providers	0	0	0	0	0	0	1	**1**	**1**
**Total**	**62**	**39**	**43**	**56**	**59**	**116**	**154**	**529**	

Policies were categorized by one investigator (VTL) when recording policies for the individual payers. To minimize subjectivity and bias, policy actions were categorized whenever possible using the original titles or formulary classifications (e.g. prior authorization for initial prescription) or intentions (e.g. feedback to providers regarding prescriptions). Another investigator (CLA) reviewed the abstracted policies for consistency in coding decisions. In the few cases of disagreement, the abstracted information was supplemented with additional information from the source document.

### Analysis

The number of actions taken by different payers and years were summarized with descriptive statistics. To examine temporal trends, Joinpoint Regression (version 4.8.0.1) [34] was used with mean number of actions by year across all eight payers as well as subgroups of top three payers, all commercial payers and Medicaid. Joinpoint Regression identifies the model with the best-fitting set of inflection points in the regression model using permutation tests [35] and calculates the annual percent change (APC) to characterize trends over time per segment. Significance tests, 95% confidence intervals (95% CI) for annual percent change and average annual percent change (AAPC) for the entire time period (if there are no inflection points identified) were also computed.

The Wayne State University Institutional Review Board concurred that the project was exempt from human subject research review.

## Results

For the eight different payers included in the analysis, there were 529 separate opioid analgesic prescription policy actions implemented across 7 years (Table 1), for a mean of 75.57 actions (95% CI 35.93, 115.22) per year. Every year had at least one policy implemented with the most actions in 2018 (*n*=154) and the fewest in 2013 (*n*=39). Of the payers analyzed, the range in number of actions implemented was wide: 36 to 103. The top three commercial payers by market share had 58, 70 and 72 total number of actions implemented while the public payer had 47 actions implemented. Not all payers implemented new opioid analgesic prescribing actions every year. There were no actions implemented by two payers in 2013, two payers in 2014 and one payer in 2015.

For the separate actions (Table 1), limitations on number of days for initial prescription (*n*=83) was the most common action followed by prior authorizations for initial prescription (*n*=77). The least common action implemented was incentives to providers which was implemented once by one payer. The public payer implemented the fewest different actions (9 of 13). Not all payers implemented every action (Table 1).

The payers differed in which actions were most commonly implemented. Three payers most frequently implemented limitations on the number of days for initial prescription, and two payers most frequently implemented prior authorizations for initial prescription. One payer most frequently implemented both limitations on the number of days for initial prescription and prior authorizations for initial prescription. One payer most frequently implemented step-edits and one payer most frequently implement limitations on number of refills and registries.

### Examples of payers having different policies addressing similar opioid prescribing challenges

To illustrate that the payers implemented separate policies, we examined their response to federal guidelines recommending opioid analgesic prescribing daily limit of ≤ 50 MME (morphine milligram equivalents) [36]. The public payer implemented a 45 MME daily limit on opioid analgesic prescriptions March 2016. One commercial payer implemented a 50 MME daily limit August 2016, one commercial payer implemented a 50 MME daily limit for short acting opioids December 2017, and one commercial payer implemented a 50 MME daily limit July 2018. The remaining four payers had limits of 90 MME or higher as of December 2018.

Another example is the response to the 2016 safety warning on combined use of opioid and benzodiazepine medications [37]. Two commercial payers implemented pharmacist safety reviews January 2017, two commercial payers implemented feedback to prescribers of opioid analgesics > 100 MME per day in combination with benzodiazepines March 2016 and June 2016, and a commercial payer implemented a registry of patients filling prescriptions for these medications June 2016. Three payers did not implement policy as of December 2018.

### Policies superseded by state law

A few policies were made moot by later state regulations. For example, three payers implemented limitations on the number of days of opioid analgesics for acute pain prior to the state implementing the same limitation by law. However, the other five payers did not implement them.

### Temporal trends

**Fig. 1:**
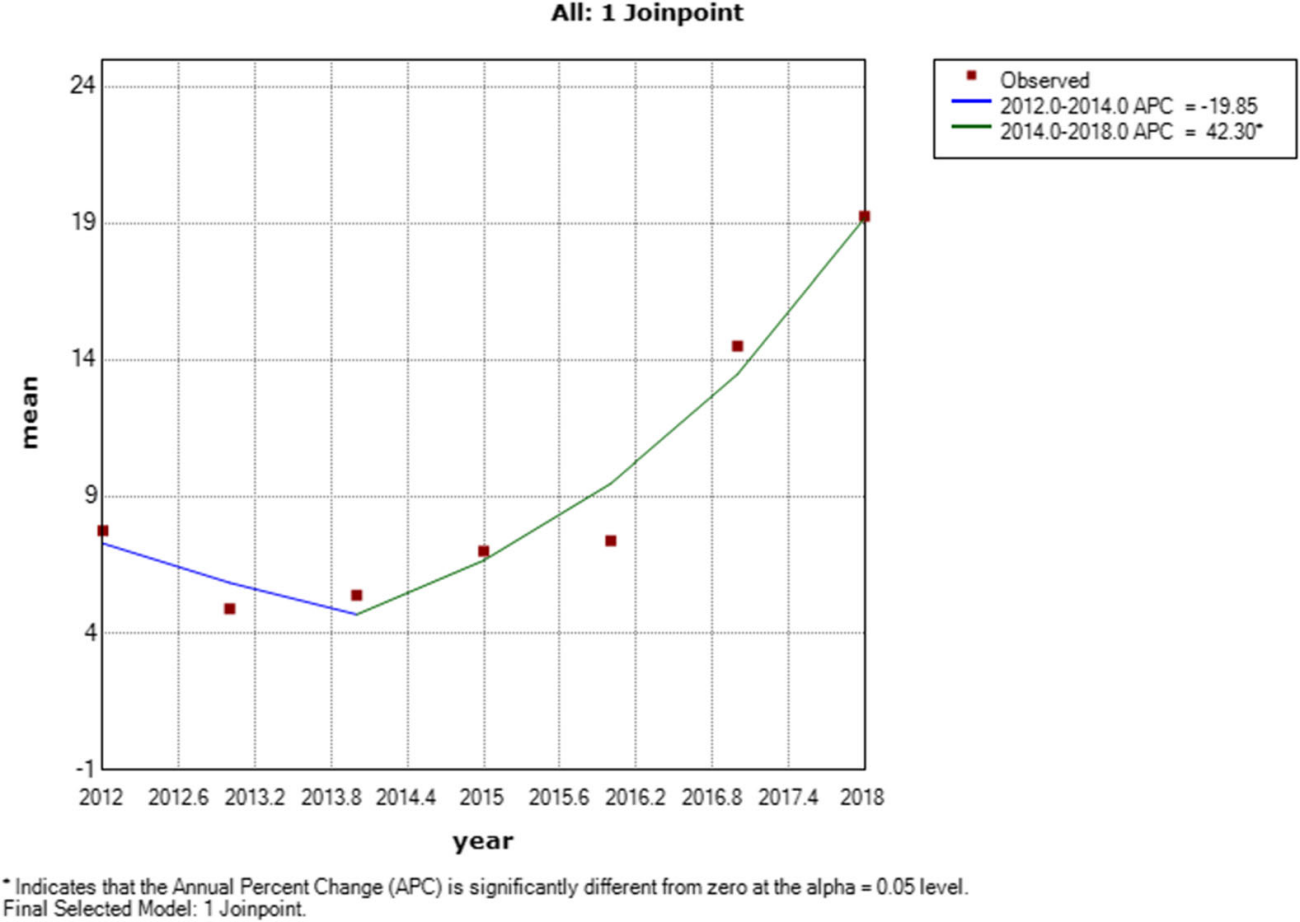
Mean number of action policies implemented by year for seven commercial and one public payer, Michigan. The line shows the average percent change with increases from 2014 to 2018

The trend line for implementing new opioid analgesic policy actions across all eight payers changed in 2014 (Fig. 1). For 2012–2014, the APC was 19.8% (not significantly different from 0) and then jumped to 42.3% for 2014–2018 (significantly different from 0, *p*<.001). Across the time period, the AAPC was 29.6% (13.2, 48.5%) for the seven commercial payers, 2.9% (− 41.6, 61.6%) for the public payer, and 23.2% (13.4, 33.9%) for the top three commercial payers in market share. No inflection point was found in subgroup analysis.

## Discussion

In this review we found commercial and public payers in Michigan implemented multiple, different opioid analgesic prescription policies during the examined period (2012–2018) that increased over time. Each payer implemented a range of policy actions during almost every year reviewed. The policies were implemented in addition to existing federal laws, Medicare regulations, and state laws and regulations on opioid prescribing. Although a few payer policies predated state law, they were in the minority. Overall, the picture was of increasingly more restrictions on opioid prescribing over time with number of policies differing sharply by payer.

In addition to the overall number of opioid analgesic prescribing policies, the payers differed in what policies actions they implemented. Limitations on number of days for initial prescription were commonly, but not universally, implemented. One commercial payer is collaborating in evaluating their limitation policy, but the analysis does not capture patients who switch carriers or pay cash [32]. Another common policy during this time period was prior authorization, a task that can be pro forma or a burdensome experience. This policy increasingly frustrates prescribers as patient access to a specific medication can be delayed or denied despite providing the required documentation [38]. Both policies are common formulary management strategies [39], and it was not surprising that they were utilized by the payers in our case study. However, there was one payer that relied heavily on step-edits, as opposed to limitations or prior authorizations, to regulate opioid prescribing.

In addition to the sheer number of policies, individually their use is not supported by an evidence-base. It is possible that some policies may improve appropriate prescribing, but it is also possible they may increase discontinuations of opioid analgesics and withholding needed medication from eligible patients with appropriate diagnoses. Other considerations include the policies impact on adherence, prescriber-patient trust, and overall utilization of healthcare resources [40]. Although the variety of policies by the different payers should offer a natural experiment to evaluate them, the sheer number of policies and the right of patients to change payers or to pay cash complicates any such analysis. We are left not knowing what strategies, or even combinations of strategies, improve prescribing practices and which ones increase patient risk for sub-therapeutic response and adverse effects. The policies also exist within the context of overlaying federal and state laws and regulations. In states without as many laws and regulations on opioid prescribing, commercial payers may implement more policies to protect enrollee safety and contain costs.

How do prescribers respond to these policies? For prescribers who accept only one insurance, such as Medicaid, the impact of all these policies is not as challenging as prescribers who accept multiple payers. Although not part of our research question, we know prescribers who are part of a team or large clinical practice that includes a pharmacist to determine the covered medications under the various payer formularies and obtains any needed prior authorization. Large clinical practices may also employ a medical assistant or utilization manager with responsibility for assisting with prior authorizations of medications. In the absence of these resources, the prescriber may have to prescribe a less effective medication or refer the patient to a pain specialist which may entail another authorization and long wait times for an appointment. The substantial time and resource commitment required by the prior authorization process alone is a factor predisposing to physician burnout [41]. In view of the increasing number of regulations, physicians may elect not to prescribe opioid or controlled substance medications. If this scenario holds, a decline in the number of prescribers would be predicted, which was in fact observed in Michigan and nationally [22].

The study is limited by time frame and examination of only one state. As Michigan has taken multiple steps to regulate opioid prescribing, analysis of policies in a less restrictive state is needed. We also did not review all commercial payers (although we included the largest carriers in the state), Medicare Advantage plans, or Michigan Medicaid managed care plans. Our review focused on commercial plan drug formularies for the commercial payers and Medicaid fee-for-service preferred drug lists. We are aware that these policies do not represent all past and present commercial payer plans. Policies may have been implemented that were missed in our retrospective review despite checking multiple sources. Other reviewers may categorize the policy actions differently. We also did not evaluate the ease of complying with policies as a prescriber, pharmacist, or patient, including the time required to obtain approval of authorization for an opioid analgesic prescription from a payer.

## Conclusion

Commercial and public payer policies on opioid analgesic prescribing must prioritize reducing diversion, misuse and overdoses through safe prescribing and dispensing practices while also prioritizing access to an essential medication class for pain management. The increase and proliferation of different action policies over time in one state that differ by payers challenges clinicians and patients to find a balance and achieve optimal clinical care for patients requiring pain management. In that respect, we concur with Comerci and colleagues [19] of finding “complicated and confusing” opioid analgesic prescribing policies.

## Data Availability

The dataset generated and analyzed during the current study are available from the corresponding author on reasonable request.

## Abbreviations

AAPC: Average annual percent change
APC: Annual percent change
CI: Confidence intervals
MME: Morphine milligram equivalents
PDMP: Prescription Drug Monitoring Program

## References

[1] National Institute on Drug Abuse (2020). National overdose deaths involving prescription opioids-number all ages 1999–2017.

[2] National Institute on Drug Abuse (2020). Decline in national overdose deaths involving prescription opioids from 2017–2018.

[3] Liu Y, Baker O, Schuur JD, Weiner SGL. Effects of rescheduling hydrocodone on opioid orescribing in Ohio. Pain Med. 2019; 10.1093/pm/pnz210. 10.1093/pm/pnz210 PMC7553017 31502638

[4] Moyo P, Simoni-Wastila L, Griffin BA, Onukwugha E, Harrington D, Alexander GC, Palumbo F (2017). Impact of prescription drug monitoring programs (PDMPs) on opioid utilization among Medicare beneficiaries in 10 US states. Addiction.

[5] Fink DS, Schleimer JP, Sarvet A, Grover KK, Delcher C, Castillo-Carniglia A, Kim J H, Rivera-Aguirre A E, Henry SG, Martins SS, Cerdá M. Association between prescription drug monitoring programs and nonfatal and fatal drug overdoses: A ystematic review. Annals of Internal Medicine, 2018168:783–790. 10.7326/M17-3074.. 10.7326/M17-3074 PMC6015770 29801093

[6] Strickler GK, Zhang K, Halpin JF, Bohnert AS, Baldwin GT, Kreiner PW (2019). Effects of mandatory prescription drug monitoring program (PDMP) use laws on prescriber registration and use and on risky prescribing. Drug Alcohol Depend.

[7] Office of Governor Rick Snyder, “Patient Protections Strengthened as State Fully Integrates MAPS into Health Systems,” 19 June 2017. http://www.michigan.gov/snyder/0,4668,7-277-73341_73343-424218%2D%2D,00.html. Accessed 18 July 2020.

[8] Al Achkar M, Grannis S, Revere D, MacKie P, Howard M, Gupta S (2018). The effects of state rules on opioid prescribing in Indiana. BMC Health Serv Res.

[9] The PEW Charitable Trusts (2016). Prescription drug monitoring programs: evidence-based practices to optimize prescriber use.

[10] The National Alliance for Model State Drug Laws (2016). Types of authorized recipients –Medicare, Medicaid, state health insurance programs, and/or health care payment/benefit provider or insurer.

[11] Haegerich TM, Paulozzi LJ, Manns BJ, Jones CM (2014). What we know, and don't know, about the impact of state policy and systems-level interventions on prescription drug overdose. Drug Alcohol Depend.

[12] Beaudoin FL, Banerjee GN (2016). Mello M J (2016). State-level and system-level opioid prescribing policies: the impact on provider practices and overdose deaths, a systematic review. J Opioid Manag.

[13] Mauri AI, Townsend TN, Haffajee RL (2020). The association of state opioid misuse prevention policies with patient- and provider-related outcomes: a scoping review. Milbank Q.

[14] Cochran G, Gordon AJ, Gellad WF, Chang CH, Lo-Ciganic WH, Lobo C, Cole E, Frazier W, Zheng P, Kelley D, Donohue JM (2017). Medicaid prior authorization and opioid medication abuse and overdose. Am J Manag Care.

[15] Howard R, Fry B, Gunaseelan V, Lee J, Waljee, Brummett C, Campbell D, Seese E, Englesbe M, Vu J (2019). Association of opioid prescribing with opioid consumption after surgery in Michigan. JAMA Surg.

[16] García MC, Dodek AB, Kowalski T, Fallon J, Lee SH, Iademarco MF, Auerbach J, Bohm MKL (2016). Declines in opioid prescribing after a private insurer policy change — Massachusetts, 2011–2015. MMWR Morb Mortal Wkly Rep.

[17] Dreyer T, Koss J, Udow-Phillips M (2015). A Tale of Three Cities: Hospital and Health System Costs in the Midwest. Center for Healthcare Research & Transformation. [Issue Brief].

[18] Roberts AW, Gellad WF, Skinner AC (2016). Lock-in programs and the opioid epidemic: a call for evidence. Am J Public Health.

[19] Comerci G, Katzman J, Duhigg D (2018). Controlling the swing of the opioid pendulum. New Engl J Med.

[20] National Institute on Drug Abuse (2020). State opioid involved overdose death rates and opioid prescribing levels.

[21] Michigan Prescription Drug Monitoring Program annual drug utilization reports, 2015-2018. https://www.michigan.gov/lara/0,4601,7-154- 89334_72600_72603_55478_55479%2D%2D-,00.html. Accessed 19 Dec 2019.

[22] Zhu W, Chernew ME, Sherry TB, Maestas N (2019). Initial opioid prescriptions among U.S. commercially insured patients, 2012-2017. New Engl J Med..

[23] U.S. Department of Health and Human Services, Prescription Drug Monitoring Program Interoperability Standards, A Report to Congress, September 2013, p4, 17–18. http://www.healthit.gov/sites/default/files/fdasia1141report_final.pdf. Accessed 31 July 2020.

[24] Michigan Public Act 248 of 2017: implemented June 1, 2018, with sanctions for failure to comply with PDMP use Public Act 249 June 1, 2018.

[25] Public Health Code Act 368 of 1978, Michigan Compiled Laws (MCL) 333.17211a.

[26] Public Health Code Act 368 of 1978 (MCL 333.1101–333.25211) amended by adding sec. 17751a. September 12, 2017. Pharmacist refusing to dispense a prescription; exempt from civil liability under certain circumstances.

[27] Michigan Public Act 246 of 2017: implemented June 1, 2018 opioid therapy consent form required for minors.

[28] Michigan Public Act 247 of 2017: implemented March 31, 2019 bona-fide established prescriber-patient relationship required to prescribe controlled substances.

[29] Michigan Public Act 251 of 2017: implemented July 1, 2018 limits opioid to a 7-day supply within a 7-day period for acute pain.

[30] Centers for Disease Control and Prevention. U.S. opioid prescribing rate maps 2006–2018. https://www.cdc.gov/drugoverdose/maps/rxrate-maps.html. Accessed 31 July 2020.

[31] Kaiser Family Foundation. Estimated based upon American Community Survey 2008–2017. www.kff.org/other/state-indicator/individual. Accessed 28 June 2020.

[32] Lin DH, Jones CM, Compton WM (2018). Prescription drug coverage for treatment of low back pain among US Medicaid, Medicare advantage, and commercial insurers. JAMA Netw Open.

[33] Samuels EA, Ross JS, Dhruva SS (2017). Medicare formulary coverage restrictions for prescription opioids, 2006 to 2015. Ann Intern Med.

[34] Joinpoint Regression Program, Version 4.8.0.1 - April 2020. Statistical Methodology and Applications Branch, Surveillance Research Program, National Cancer Institute.

[35] Kim HJ, Fay MP, Feuer EJ, Midthune DN (2000). Permutation tests for joinpoint regression with applications to cancer rates. Stat Med.

[36] Dowell D, Haegerich TM, Chou R. CDC guideline for prescribing opioids for chronic pain — United States, 2016. MMWR Recomm Rep. 2016;65(No. RR-1):1–49. 10.15585/mmwr.rr6501e1. (correction: 2016;65:295). 10.15585/mmwr.rr6501e1 26987082

[37] U.S. Food and Drug Administration. Drug Safety Communications. FDA warns about serious risks and deaths when combining opioid pain or cough medicine with benzodiazepines; requires its strongest warning. August 31, 2016. https://www.fda.gov/media/99761/download Accessed 30 July 2020.

[38] Salam T, Duhig A, Patel AA, Cameron A (2020). Physicians’ perspectives regarding non-medical switching of prescription medications: Results of an internet e-survey. PLoS One.

[39] Department of Health & Human Services. Pharmacies Formularies Coverage rules. https://www.medicare.gov/Pubs/pdf/11136-Pharmacies-Formularies-Coverage-Rules.pdf. Accessed 10 Dec 2020.

[40] Nguyen E, Weeda ER, Sobieraj DM, Bookhart BK, TakPiech C, Coleman CI (2016). Impact of non-medical switching on clinical and economic outcomes, resource utilization and medication-taking behavior: a systematic literature review. Curr Med Res Opin.

[41] A Crisis in Health Care: A Call to Action on Physician Burnout. Massachusetts Medical Society, Massachusetts Health and Hospital Association, Harvard T.H. Chan School of Public Health, and Harvard Global Health Institute. 2018. http://www.massmed.org/News-and-Publications/MMS-News-Releases/Physician-Burnout-Report-2018/ Accessed 11 Dec 2020.

